# A bio-inspired swarm UAV framework integrating thermal sensing and optimization-based coordination for efficient search and rescue operations

**DOI:** 10.1038/s41598-025-33223-z

**Published:** 2025-12-27

**Authors:** Abbas Aqeel Kareem, Ahmed Jabbar Abid, Dalal Abdulmohsin Hammood, Raaid Alubady, Salam J. Yaqoob, Omar Almomani, Oleksandr Rubanenko

**Affiliations:** 1 Electrical Engineering Technical College, Middle Technical University, Baghdad, Iraq; 2 Technical Engineering College of Artificial Intelligence, Department of Cybersecurity Engineering Techniques, Middle Technical University, Baghdad, Iraq; 3 Department of Information Networks, College of Information Technology, University of Babylon, Babel, Iraq; 4 Al-Ayen Iraqi University, Thi-Qar, Iraq; 5 Training and Energy Researches Office, Ministry of Electricity, Thi-Qar, 64001 Iraq; 6 Department of Networks and Cybersecurity, Hourani Center for Applied Scientific Research (HCASR), Al-Ahliyya Amman University, Amman, Jordan; 7 Department of Power Plants and System , Vinnytsia National Technical University , Vinnytsia, 21000 Ukraine

**Keywords:** Bio-inspired optimization, Exploration score, Multi-UAV systems, Search and rescue, Swarm intelligence, Thermal imaging, UAV coordination, Unmanned aerial vehicles, Engineering, Mathematics and computing

## Abstract

Search and rescue (SAR) operations demand rapid, reliable detection of survivors in disaster-stricken environments where time, terrain, and responder safety are critical constraints. While Unmanned Aerial Vehicles (UAVs) equipped with thermal imaging offer a promising aerial solution, current approaches often struggle to balance large-area coverage, responsiveness to thermal cues, and avoidance of redundant search paths. This paper proposes a modular, bio-inspired swarm UAV framework that enables real-time thermal-based SAR through autonomous, cooperative exploration of a discretized search grid. Each UAV operates as an intelligent agent, leveraging bio-inspired optimization algorithms to determine its next target location, with decision-making grounded in a shared thermal confidence map. A novel Exploration Score metric is introduced to quantitatively assess search efficiency by integrating area coverage, redundancy minimization, and spatial dispersion of the swarm. The system is implemented in a high-fidelity PX4 + Gazebo simulation environment, featuring real-time thermal detection and multi-drone coordination. Ten bio-inspired algorithms are evaluated under identical conditions, with Particle Swarm Optimization (PSO) achieving the highest exploration score of 0.67, outperforming Grey Wolf Optimizer (0.62) and Ant Colony Optimization (0.59). PSO also achieved 80% area coverage within 60% of the mission time while maintaining a low redundancy ratio (~ 0.25) and balanced inter-drone separation (15–20 m). These results confirm the framework’s effectiveness in enabling fast, adaptive, and energy-efficient thermal search missions, significantly enhancing the probability of early survivor detection. The proposed architecture serves as a benchmarking platform for UAV swarm coordination, and the exploration score metric offers a unified performance measure for future SAR algorithm comparisons.

## Introduction

Natural catastrophes, such as earthquakes and wildfires, and man-made disasters, such as nuclear meltdowns and other industrial accidents, killed more than 1.5 million people between 2000 and 2014^1^. There is an urgent need for technology that can aid in disaster recovery as the world prepares for an increase in the frequency of catastrophic events, such as hurricanes, and as the globe’s population density rises, potentially leading to a higher number of fatalities^2^. Technology that can aid in disaster recovery is desperately needed. Almost any kind of disaster is seen as benefiting from the employment of robots. They might be used in the future as human stand-ins who can enter dangerous environments, as supplements to enhance human feelings and manipulation skills, or as explorers in environments that are inaccessible to humans. Robots have been used in disaster response since 2001, following decades of advancements in robotic platform development^3^. Recent works demonstrate the increasing integration of UAVs with advanced sensing payloads for disaster robotics, particularly in thermal-based detection and real-time decision-making^4–6^. Furthermore, UAV swarms are now being evaluated in simulation and real-world trials, confirming their superior responsiveness and coverage in disaster scenarios^7^.

UAVs’ excellent mobility, cheap maintenance costs, ease of deployment, and hovering capability make them suitable for a wide range of civil applications^8^. Real-time road traffic monitoring, wireless coverage, remote sensing, search and rescue, cargo delivery, security and surveillance, precision agriculture, and civil infrastructure inspection are all applications for these vehicles. Using a swarm of UAVs rather than a single UAV can provide several general benefits for tasks that are either impossible for a single robot to complete or that require assistance from a multi-robot arrangement. Swarm UAV teams’ primary benefits include shorter task execution times, enhanced resilience, redundancy, and fault tolerance, as well as the ease of cooperative capabilities such as dynamic collaboration (e.g., localizing ionizing radiation sources)^9^, distributing the application payload, and dynamic collaboration (e.g., cooperative object transport^10^. So the recent studies confirm that swarm-based UAV frameworks improve mission scalability and adaptability, addressing key issues such as communication robustness and cooperative decision-making^11,12^.

The COMETS project (real-time coordination and control of multiple heterogeneous unmanned aerial vehicles) demonstrated an early method for designing and developing heterogeneous multi-UAV systems for cooperative activities^13^. The project’s goal was to design and implement a distributed control system for collaborative operations with heterogeneous UAVs. To do this, the project researchers created an autonomous helicopter and a remote-controlled airship and worked toward real-time cooperative perception^13–15^.

Regarding human-robot cooperation for SAR operations, PeLoTe^16^ One of the first EU-funded projects in SAR robotics created a heterogeneous telematic system for cooperative (human-robot) SAR operations and produced mobile robots for SAR missions. Other international programs, such as the NIFTi EU project (natural human-robot interaction in dynamic environments), are creating and developing autonomous multi-robot systems for SAR missions, ICARUS (Integrated Components for Assisted Rescue and Unmanned Search operations)^17,18^, TRADR (long-term human-robot teaming for disaster response)^19–21^, or SmokeBot (mobile robots with novel environmental sensors for inspection of disaster sites with low visibility)^22,23^.

Other projects, such as CENTAURO (robust mobility and dexterous manipulation in disaster response by full-body telepresence in a centaur-like robot), have focused on the development of more advanced robots that are not fully autonomous but controlled in real time^24^. Both UGVs and UAVs were used in NIFTi for mapping and autonomous navigation in challenging conditions^25^. The project’s primary focus was on human-robot interaction and information distribution for human operators at various operational levels. In a similar vein, the TRADR project focused on multi-robot planning and human-robot collaboration in disaster response^19–21^. A framework for integrating UAVs in SAR missions, from path planning to a global 3D point cloud generator, is specifically one of TRARD’s outputs^26^. In keeping with the concept, the German Rescue Robotics Center was established at Fraunhofer FKIE, where more extensive research is carried out, including maritime SAR^27^. For quick deployment, ICARUS project researchers created a set of UAVs, USVs, a huge UGV, and an unmanned marine capsule that functions as a UUV. Additionally, a multi-domain robot command and control station, middleware software for tactical communications, and mapping tools^28^. While the architecture of multi-robot systems and the algorithmic aspects of SAR operation were the primary focus of these studies, Smokebot concentrated on creating sensors and sensor fusion techniques for challenging situations^22,23^. Some of these initiatives, particularly those that began before 2017, are described in further depth in^28^. Recent studies have explored challenges in multi-UAV deployment for SAR, including human-swarm interaction, system scalability, and communication robustness^29^. The authors in^30^ highlighted the role of semi-voluntary human-robot cooperation in swarm SAR operations, while the study in^31^ Proposed resilient swarm protocols to recover isolated agents during dynamic missions. More recently, projects have expanded to autonomous maritime SAR and AI-assisted aerial robotics, where UAVs cooperate with other robotic platforms to enhance multi-domain operations^32,33^. These efforts reflect a broader trend toward swarm robotics in SAR, yet al.so highlight limitations in scalability and algorithmic generalization. Table 2 summarizes and compares 15 major SAR projects with the proposed framework, highlighting key differences in focus, strengths, and limitations.

While prior projects have laid a solid foundation for multi-UAV systems in search and rescue (SAR), a critical gap remains in scalable frameworks that integrate optimization-based coordination with real-time thermal sensing and distributed intelligence. Specifically, many earlier efforts lack the flexibility to test and compare a wide variety of decision-making algorithms under the same system constraints. Furthermore, existing systems often focus on either human–robot interaction, hardware design, or low-level sensing, without systematically addressing the trade-off between search efficiency, response time, and redundancy avoidance. To address these limitations, this study proposes a modular framework for multi-drone thermal search based on bio-inspired optimization algorithms, such as Particle Swarm Optimization (PSO)^34^, Ant Colony Optimization (ACO)^35^, Grey Wolf Optimization (GWO)^34^, and others. Alternative optimization strategies such as PPSwarm^35^, which integrates RRT* with PSO, and exact MIP-based coverage algorithms^36^, have shown strong results in constrained SAR scenarios. A* variants have also been used to optimize path lengths and reduce computational overhead^37^. Recent comparative analyses further demonstrate the role of novel swarm-inspired methods, including pigeon-inspired optimization, snow-ablation strategies, and enhanced grey wolf optimizers, in achieving efficient path planning under dynamic and uncertain conditions^38–41^. Surveys confirm the dominance of bio-inspired approaches in UAV path optimization, emphasizing their suitability for large-scale cooperative SAR missions^42,43^. However, recent advances in UAV object detection and deep-learning–based thermal recognition have significantly improved accuracy and reliability^44^. These advances enable UAV swarms not only to explore efficiently but also to detect survivors with higher confidence, reinforcing their role as an indispensable tool for modern SAR missions. Recent research has increasingly explored adaptive learning and decentralized coordination for UAV-based SAR operations. For example, adaptive Q-learning methods have been proposed to improve path planning efficiency in unknown environments, enabling faster convergence and autonomous policy adaptation^45^. Similarly, UAV-based real-time survivor detection systems integrate onboard perception with deep models for immediate post-disaster response. In contrast, decentralized deep reinforcement learning frameworks have been employed for distributed scheduling and cooperative task allocation in dynamic multi-agent systems^46,47^. These studies provide complementary perspectives on adaptive control, real-time perception, and distributed optimization that align with and motivate future extensions of the present coverage-based framework toward learning-enabled and decentralized SAR coordination.

Unlike previous projects summarized in Table 1, which emphasized hardware integration or human–robot interaction, this framework provides the first unified benchmarking of swarm optimization strategies combined with real-time thermal detection. In this work, each drone runs as an autonomous agent, sharing a global thermal confidence map and dynamically selecting its next position based on a discrete grid model. A novel exploration score metric is introduced to evaluate search performance. Our method is implemented in a realistic simulation environment using PX4 + Gazebo, with multiple UAVs running concurrently and evaluating different search strategies.

This study focuses exclusively on the high-altitude phase of thermal search, where drones operate at safe survey altitudes to rapidly assess large areas using thermal imagery. Through extensive experimentation with 10 optimization algorithms, we prove that PSO achieves an exploration score of 0.67, highlighting its effective balance between search breadth and detection responsiveness. The main contributions of this work are summarized as follows:

1. A fully integrated, multi-drone thermal search framework supporting parallel deployment of metaheuristic-based agents.
2. Introduction of a novel exploration score metric to objectively compare search performance across different algorithms.
3. Real-time integration of thermal detection with coordinated area exploration using a shared grid-based representation.
4. A comparative evaluation of 10 bio-inspired optimization algorithms, with detailed performance insights under identical operational conditions.

The rest of the article is included in Sect. “Methodology,” the proposed method, including the grid-based thermal search strategy, the definition of the exploration score metric, and the integration of metaheuristic algorithms for drone coordination. Section 3 describes the system implementation, including the simulation environment, UAV configuration, communication interface, and real-time thermal detection pipeline. Section 4 reports the results and discussion, providing quantitative comparisons of 10 bio-inspired optimization algorithms based on exploration performance. Finally, Sect. 5 concludes the paper, summarizing the key findings and outlining future directions for extending the framework to incorporate additional search phases and facilitate real-world deployment.

**Table 1:** Major international search and rescue (SAR) robotics projects.

Project	Main idea	Strengths	Limitations	References
COMETS	Multi-UAV coordination for cooperative fire detection	Early demonstration of heterogeneous UAVs; real-time control	Centralized, limited scalability, and optimization	^13^
PeLoTe	Human–robot cooperation in SAR	Integration of human operators with robots	Minimal autonomy, limited scalability	^16^
NIFTi	Human–robot interaction in dynamic SAR	Mapping, autonomy in UGV + UAV teams	Focused on HRI, less on UAV swarm optimization	^48^
ICARUS	Maritime & aerial SAR platforms (UAVs, UGVs, USVs, UUVs)	Multi-domain platforms, strong hardware	Limited algorithmic evaluation, centralized coordination	^49^
TRADR	Long-term human–robot teaming for disaster response	Persistent missions, multi-robot planning	Weak UAV swarm optimization, UGV focus	^50^
SmokeBot	Sensor fusion in low-visibility sites	Novel radar/LiDAR sensing	Focused on UGVs, limited UAV swarms	^51^
CENTAURO	Full-body telepresence for disaster response	Robust mobility, dexterous manipulation	Tele-operated, not swarm-based	^24^
SHERPA	Heterogeneous UAV + UGV teams for alpine SAR	Cooperation in complex terrains	Limited scalability, complex integration	^52^
euRathlon	Pan-European robotics competitions	Benchmarking in realistic disaster scenarios	Focus on competitions, not algorithmic studies	^18^
NIFTy-DR	UAV-based disaster information gathering	Enhanced autonomy and mapping	Extension of NIFTi, still HRI-centric	^53^
DARIUS	Deployable SAR for CBRN incidents	Integration of robotics with disaster management	More on system deployment than UAV autonomy	^54^
INACHUS	SAR in collapsed buildings	Advanced sensing, situational awareness	More UGV-based, limited UAV swarm	^55^
CURSOR	UAV swarms with sensors for rubble search	Real UAV swarms, advanced sensors	Limited optimization strategy comparison	^56^
RoboCup Rescue/RobOrder	SAR competitions & benchmarks	Global community, standard testing	Simulation-driven, not UAV swarm-focused	^57^
DRZ (German Rescue Robotics Center)	National testbed for SAR robotics	Integration of UAVs and ground robots in testing	Still experimental, more system validation than algorithms	^58^
This Work	Bio-inspired UAV swarm for thermal SAR	Evaluation of bio-inspired algorithms with real-time thermal integration	Current validation in simulation only	–

## Methodology

The proposed method presents a modular framework for high-altitude thermal search using a swarm of drones coordinated by bio-inspired optimization algorithms. This framework enables each UAV to operate autonomously within a discretized search area, utilizing real-time thermal sensing to detect human presence and employing dynamic optimization techniques to coordinate exploration. The system uses a distributed decision-making approach, where the search area is first divided into grid cells. Each UAV is tasked with navigating to unexplored regions while minimizing redundancy and maximizing coverage.

The overall search process is divided into two primary components:

1. The ground station side, responsible for managing the global search map and computing the best following locations for each UAV using bio-inspired algorithms.
2. The drone side, where each UAV autonomously navigates to its assigned location, captures thermal imagery, processes the data to estimate the probability of human presence, and communicates the results back to the ground station.

**Fig. 1:**
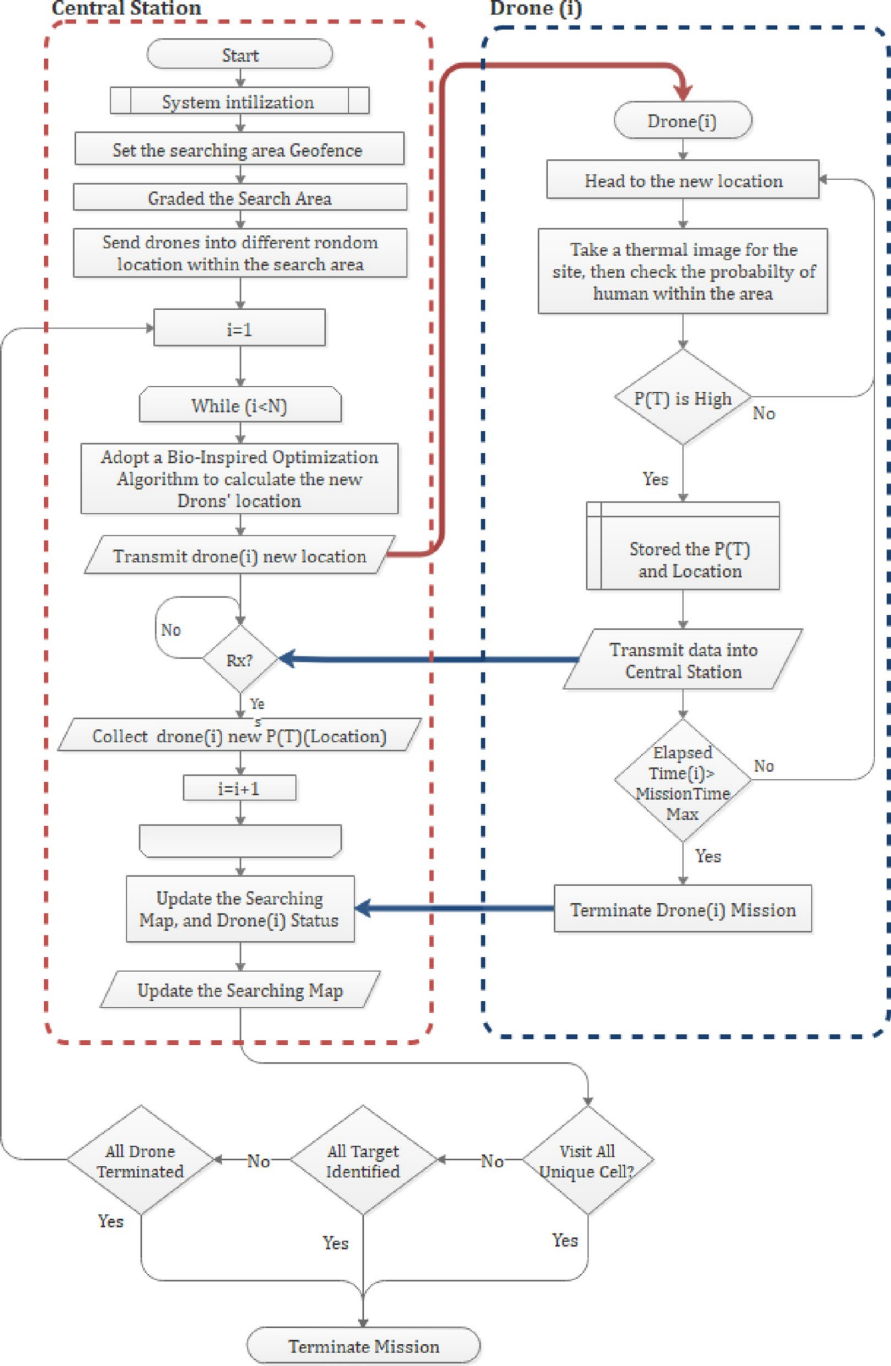
Multi-UAV thermal-based search flowchart.

The sequential flow of operations between these two components is summarized in Fig. 1, which outlines the main stages of the high-altitude thermal search mission from system initialization to mission termination.

### Grid division strategy

To ensure full aerial coverage of the search area, the entire region is discretized into uniform grid cells based on the drone’s altitude and camera field of view. This transformation converts a continuous GPS-defined region into a structured environment for discrete search and decision-making. Algorithm 1 describes the Grid Division Strategy.

**Fig. 2:**
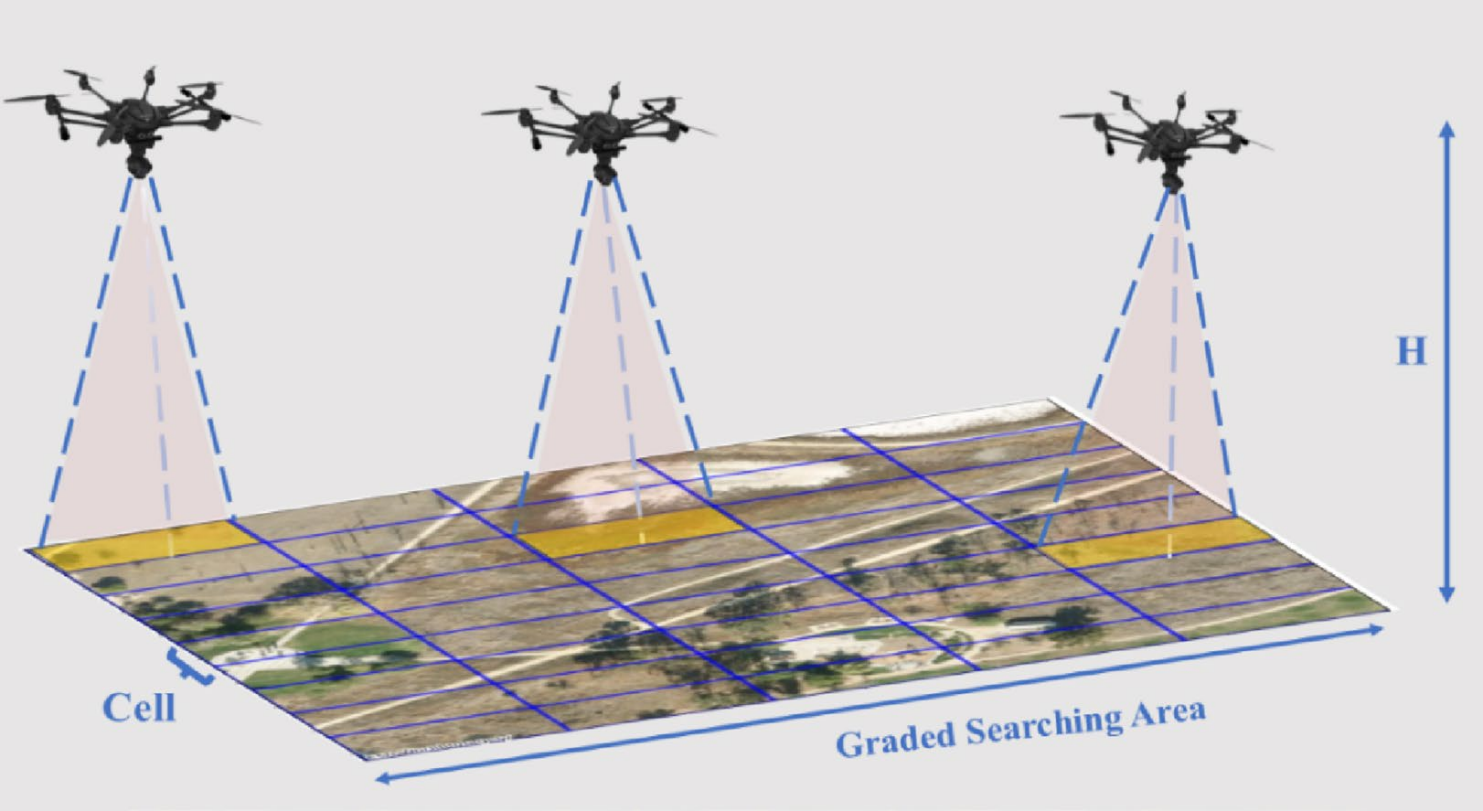
High-Altitude search coverage model for a swarm of drones: illustration of ground area division and individual drone field-of-view.

Each grid cell is then represented by its center GPS coordinate, which is mapped from its corresponding grid index using a predefined origin and cell resolution. This enables correct conversion between discrete decision-making and GPS-based actuation. The final grid is a matrix, where each entry holds the state of exploration and potential thermal detection confidence. As illustrated in Fig. 2, the graded searching area is divided into multiple grid cells, with each drone covering a subset of the area from a fixed altitude. The drones’ sensors project downward, scanning their assigned cells as they follow the search mission plan. This spatial division facilitates optimal area allocation among swarm members, minimizes overlap, and enables the dynamic reassignment of unexplored cells during mission execution.

**Figure:**
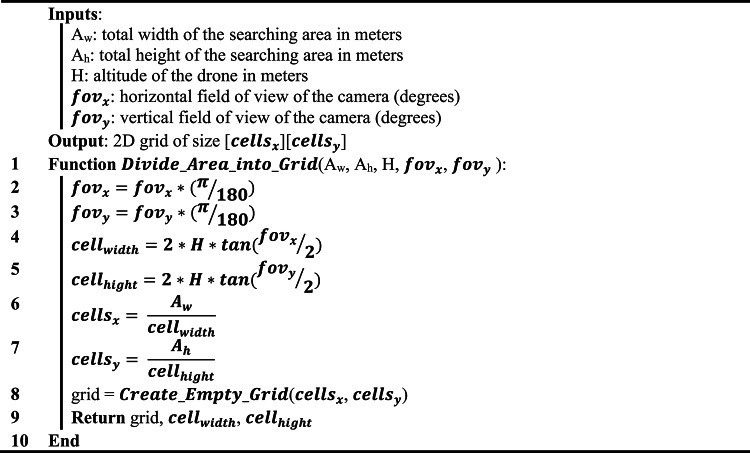
Algorithm 1: Divide the search area into grid cells

### Bio-Inspired optimization algorithms

Bio-inspired optimization algorithms are computational methods inspired by natural processes such as animal behavior, evolution, and social interactions. These algorithms utilize simple rules observed in nature—such as swarm movement, predator-prey dynamics, or genetic inheritance—to solve complex optimization problems. They are especially effective in dynamic and uncertain environments, making them ideal for applications like autonomous drone exploration, where adaptability, collaboration, and efficient decision-making are crucial. Table 2 compares several bio-inspired optimization algorithms used in autonomous drone exploration, focusing on their natural inspiration, key mechanisms, and balance between exploration and exploitation. Algorithms such as CS, GA, and ABC offer strong global search capabilities, while PSO, GWO, and WOA excel at refining existing solutions. Others, such as DOA and BA, keep a balanced approach. This comparison helps guide the selection of suitable algorithms based on mission needs, such as wide-area search or local precision.

The objective of optimizing all algorithms in this study is to minimize the mission cost function, which balances temporal efficiency and detection effectiveness. Specifically, the objective function seeks to maximize the coverage of victim-suspected cells while minimizing total flight path length and mission duration, expressed as (1):

1 $$\:\mathrm{min}f\left({t}_{1},\:{t}_{2},\:\dots\:.\:,\:{t}_{n}\right)=\:\frac{{D}_{C}}{\raisebox{1ex}{$h$}\!\left/\:\!\raisebox{-1ex}{$v$}\right.}\:\:$$

where $\:h\:$ represents the Euclidean distance between consecutive cells, $\:v\:$ is the UAV’s constant velocity, and $\:{D}_{C}$ Denotes the detection confidence associated with the thermal response at the current cell. The optimization algorithm thus determines the optimal sequence of waypoints $\:({t}_{1},{t}_{2},\dots\:,{t}_{n})$ that achieves the best trade-off between search efficiency and detection reliability.

For all metaheuristic algorithms, candidate solutions are represented as vectors of decision variables corresponding to the spatial coordinates of UAV waypoints within the exploration grid. Each individual thus encodes a potential sequence of target cells defining the flight path for a single UAV. The optimization process updates these position vectors iteratively to minimize the objective function described in (1). For example, in the case of the Genetic Algorithm (GA), each chromosome encodes the ordered sequence of visited cells, with crossover and mutation operations applied to exchange and perturb waypoint positions, respectively. This representation allows direct mapping between optimization outputs and UAV trajectories, ensuring comparability across all algorithms.

### Thermal detection strategy

**Fig. 3:**
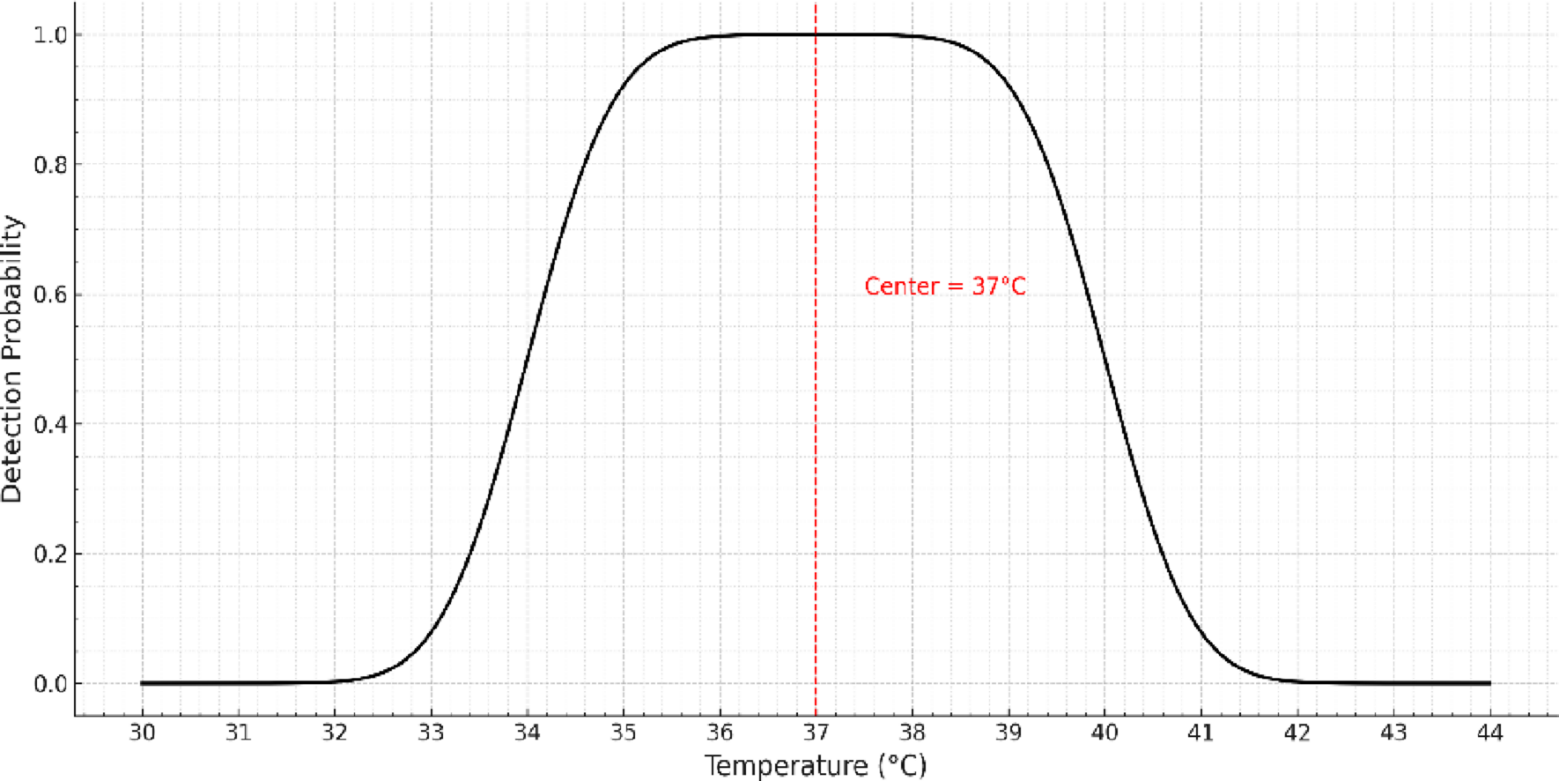
Flattened top-hat probability function for human detection.

Thermal target detection plays a crucial role in locating potential survivors during search-and-rescue missions, particularly in conditions where visual cues are limited. In this work, each drone is equipped with a downward-facing thermal camera, capable of capturing real-time infrared imagery during flight. The core thermal detection process relies on analyzing pixel intensities within the infrared image frame. Human bodies and warm-blooded animals emit infrared radiation typically within a distinct temperature range. These temperatures manifest as high-intensity regions in the thermal image, which allows the system to infer the likelihood of human presence. Thermal target detection is enhanced through a smoothed top-hat probability model. As shown in Fig. 3, the flattened top-hat function assigns a maximum detection probability to temperatures in the typical human body range (36–38 °C), with smooth transitions outside this range to avoid abrupt detection thresholding. The human detection probability based on temperature is modeled using a smoothed top-hat function^68^, denoted as $\:\boldsymbol{P}(\boldsymbol{T};\boldsymbol{L},\boldsymbol{T}\boldsymbol{c},\boldsymbol{W},\boldsymbol{S})$, where $\:\boldsymbol{T}$ represents the measured temperature, $\:{\boldsymbol{T}}_{\boldsymbol{C}}$​ is the center temperature corresponding to typical human body temperature, $\:\boldsymbol{W}$ Is the width of the flat detection region, and $\:\boldsymbol{S}\:$Is the smoothing width applied at the boundaries. The function is defined as (2):

2 $$\:\boldsymbol{P}\left(\boldsymbol{T};\boldsymbol{L},\:{\boldsymbol{T}}_{\boldsymbol{c}},\:\boldsymbol{W},\:\boldsymbol{S}\right)=\left\{\begin{array}{c}H(\frac{\boldsymbol{T}-{\boldsymbol{T}}_{\boldsymbol{m}\boldsymbol{i}\boldsymbol{n}}}{\boldsymbol{S}};L),\:\:T\in\:[{\boldsymbol{T}}_{\boldsymbol{m}\boldsymbol{i}\boldsymbol{n}}-S,\:{\boldsymbol{T}}_{\boldsymbol{m}\boldsymbol{i}\boldsymbol{n}}]\\\:1,\:\:\:\:\:\:\:\:\:\:\:\:\:\:\:\:\:\:\:\:\:\:\:\:\:\:\:\:\:\:\:\:\:\:\:\:T\in\:\left[{\boldsymbol{T}}_{\boldsymbol{m}\boldsymbol{i}\boldsymbol{n}},\:{\boldsymbol{T}}_{\boldsymbol{m}\boldsymbol{a}\boldsymbol{x}}\right]\\\:H(\frac{{\boldsymbol{T}}_{\boldsymbol{m}\boldsymbol{a}\boldsymbol{x}}-\boldsymbol{T}}{\boldsymbol{S}};L),\:\:T\in\:[{\boldsymbol{T}}_{\boldsymbol{m}\boldsymbol{a}\boldsymbol{x}},\:{\boldsymbol{T}}_{\boldsymbol{m}\boldsymbol{a}\boldsymbol{x}}+S]\end{array}\right.$$

where the parameters $\:{\boldsymbol{T}}_{\boldsymbol{m}\boldsymbol{i}\boldsymbol{n}},\:{\boldsymbol{T}}_{\boldsymbol{m}\boldsymbol{a}\boldsymbol{x}}$ are defined as (3):

3 $$\:{\boldsymbol{T}}_{\boldsymbol{m}\boldsymbol{i}\boldsymbol{n}}=\:{\boldsymbol{T}}_{\boldsymbol{C}}-\frac{\boldsymbol{W}}{2},\:\:{\boldsymbol{T}}_{\boldsymbol{m}\boldsymbol{a}\boldsymbol{x}}=\:{\boldsymbol{T}}_{\boldsymbol{C}}+\frac{\boldsymbol{W}}{2}$$

The function is a smooth transition function, typically based on the error function^68^, and is expressed as (4):

4 $$\:\mathcal{H}\left(\boldsymbol{y};\boldsymbol{L}\right)=\:\frac{1}{2}[1+\mathbf{erf}\left(\frac{\boldsymbol{L}.\:\boldsymbol{y}}{\sqrt{1-{\boldsymbol{y}}^{2}}}\right)$$

Here, $\:\boldsymbol{L}$ It is a shape parameter that controls the sharpness of the transition between the flat region and the tails, and $\:\boldsymbol{e}\boldsymbol{r}\boldsymbol{f}(\cdot\:)$ Is the standard error function.

The flattened top-hat probability function models human detection based on temperature by assigning a maximum constant probability within a central flat region corresponding to typical human body temperatures, reflecting a high likelihood of human presence. In the adjacent transition regions, the probability decreases smoothly using error-function-based smoothing, which prevents abrupt changes and ensures stable detection near the region boundaries. Outside these smoothed regions, the probability rapidly approaches zero, showing a negligible likelihood of detecting a human at those temperature values.

**Table 2:** Bio-Inspired algorithms comparison.

Algorithm	Inspiration	Key mechanisms	Exploration capability	Exploitation capability	Exploration/Exploitation tunning capability
Particle Swarm Optimization (PSO)^59^	Swarm behavior of birds/fish	Velocity and position updates based on personal and global best positions	Moderate	Strong	Yes (by$\:w,\:c1,\:c2$)
Grey Wolf Optimizer (GWO)^34^	Grey wolves’ hunting strategy	Hierarchical role-based movement: α, β, δ guide others; encircling and attacking prey	Moderate	Strong	Yes (by$\:a$)
Cuckoo Search (CS)^60^	Cuckoo’s parasitic egg-laying	Lévy flights; survival of best nests; discovery probability	Strong	Moderate	Yes (by$\:\rho\:,\:{a}_{s}$)
Ant Colony Optimization (ACO)^61^	Ants’ pheromone-based foraging	Pheromone deposition and evaporation; probabilistic path choice	Moderate	Strong	Yes (by$\:\alpha\:,\:\beta\:,\:\rho\:$)
Dolphin Optimization Algorithm (DOA)^62^	Echolocation and cooperative hunting	Sonar exploration, subgroup cooperation, and information sharing	Strong	Strong	Yes (by$\:P{P}_{1},\:{R}_{e}$)
Crow Search Algorithm (CSA)^63^	Crows’ food caching and deception	Memory-based tracking, awareness probability APAP, random relocation	Strong	Moderate	Yes (by$\:AP,\:FL$)
Genetic Algorithm (GA)^64^	Darwinian evolution	Choice, crossover, mutation	Strong (via mutation)	Strong (via choice)	Yes (by$\:{P}_{c},\:{P}_{m}$)
Whale Optimization Algorithm (WOA)^65^	Humpback whales’ bubble-net hunting	Spiral motion, encircling prey, random search	Moderate	Strong	Yes (by$\:b,\:a$)
Bat Algorithm (BA)^66^	Bats’ echolocation	Frequency tuning, loudness attenuation, pulse adaptation	Moderate to Strong	Strong	Yes (by $\:{f}_{min},\:{f}_{max}\:,\:r,\:A$)
Artificial Bee Colony (ABC)^67^	Foraging behavior of honeybees	Roles of employed, onlooker, and scout bees; solution abandonment and recruitment	Strong	Moderate	Abandonment limit)

## Design and simulation

The proposed multi-drone thermal search system was implemented using a high-fidelity simulation environment that replicates real-world aerial search-and-rescue conditions. The system comprises several integrated modules, each responsible for specific functionalities, including drone control, thermal data acquisition, metaheuristic-based navigation, and mission coordination.

### Simulation environment

The drones run within a simulated environment built using Gazebo and PX4 autopilot firmware. Each drone is equipped with:

1. A downward-facing simulated thermal camera.
2. MAVSDK-based autonomous flight control interface.
3. GStreamer-based real-time video streaming.

**Fig. 4:**
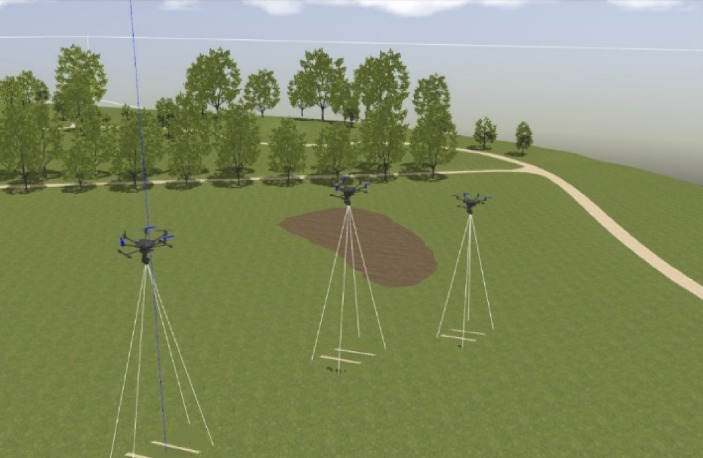
Simulated deployment of three UAVs in a Gazebo environment performing high-altitude search with downward-facing cameras.

The search area is defined as a two-dimensional region and discretized into uniform grid cells (e.g., 30 × 60 m per cell for high-altitude flights at 25 m, as determined from camera field-of-view calculations). The grid enables structured decision-making and coverage analysis. Figure 4 illustrates the simulated deployment of three UAVs within the Gazebo simulation environment, performing a high-altitude thermal search mission using downward-facing cameras. This simulation setup replicates a realistic aerial search-and-rescue (SAR) scenario, where each UAV autonomously navigates the search grid while keeping the best altitude and camera orientation.

To maintain consistency across all tested algorithms, the parameter settings were primarily adopted from their standard formulations in the literature and adjusted only through limited preliminary trials to ensure stable convergence in our simulation environment. No extensive parameter tuning or meta-optimization was applied, as the main objective of this study was to compare the algorithms’ relative coordination performance under identical mission conditions rather than to achieve their individual global optima. Table 3 lists the specific parameter values used in the experiments.

### Drone control and path execution

Each UAV was controlled via the MAVSDK-Python API, which enabled asynchronous waypoint navigation, telemetry monitoring, and mission management. The core mission loop ensured that each drone:

1. Received its next waypoint from its metaheuristic algorithm.
2. Navigated autonomously to that location using GPS coordinates.
3. Captured and processed thermal imagery upon arrival.
4. Updated its local memory and shared global map.

Control commands, such as takeoff, waypoint tracking, and return-to-home, were implemented using thread-safe routines to handle multiple drones in parallel.

In the current implementation, all UAVs operate at a uniform altitude during the high-altitude search phase to ensure consistent sensor footprints and simplify grid-based coverage analysis. As such, no explicit inter-drone collision-avoidance mechanism is incorporated in this version of the framework. Although the grid allocation and waypoint separation inherently minimize overlap in practice, full three-dimensional collision-free navigation will be addressed in future extensions through altitude-layer assignment or distributed avoidance strategies (e.g., potential-field or velocity-obstacle models).

It should be noted that the proposed framework addresses a coverage-based search strategy rather than a probabilistic or moving-target formulation. The objective is to achieve complete and efficient area coverage to identify static, victim-suspected thermal regions, assuming no temporal motion of targets during the mission. This contrasts with probabilistic or dynamic search paradigms, which explicitly model temporal target displacement and uncertainty. By focusing on coverage-driven coordination, the present method emphasizes spatial efficiency, redundancy minimization, and cooperative path planning under static environmental conditions.

**Table 3:** Parameter settings of the bio-inspired optimization algorithms.

Algorithm	Parameter name	Symbol	Value/Range
PSO	Inertia weight	$\:\omega\:$	0.9
	Cognitive coefficient	$\:{c}_{1}$	1.5
	Social coefficient	$\:{c}_{2}$	1.9
GWO	Convergence constant	$\:a$	Linearly decreases 2 → 0
CS	Discovery rate	$\:\rho\:$	0.2
	Lévy flight step size	$\:{a}_{s}\:$	1.5
ACO	Pheromone importance	$\:\alpha\:$	1.0
	Heuristic importance	$\:\beta\:$	2.0
	Evaporation rate	$\:\rho\:$	0.5
DOA	Initial convergence factor	$\:P{P}_{1}$	0.10–0.15
	Effective radius	$\:{R}_{e}$	0.25
CSA	Awareness probability	$\:AP$	0.1
	Flight length	$\:FL$	2.0
GA	Crossover probability	$\:{P}_{c}$	0.7
	Mutation probability	$\:{P}_{m}$	0.1
WOA	Spiral constant	$\:b$	1.0
	Convergence constant	$\:a$	Linearly decreases 2 → 0
BA	Frequency range	$\:{f}_{min},\:{f}_{max}\:\:$	(0.0, 2.0)
	Pulse rate	$\:r$	0.5
	Loudness	$\:A$	1.0
ABC	Employed/onlooker ratio		1 : 1
	Abandonment limit		5 trials

### Thermal target detection

Each drone performed thermal target detection by processing live image frames streamed via the GStreamer protocol. The detection module computed a thermal confidence score for each grid cell based on pixel intensity analysis. If the normalized pixel intensity exceeded a predefined threshold, the cell was flagged as a potential human location.

Thermal confidence values were stored in a shared map accessible by all agents, allowing collaborative decision-making and global coordination. High-confidence detections were logged, including geolocation and timestamp, for evaluation.

### Ground control and visualization

**Fig. 5:**
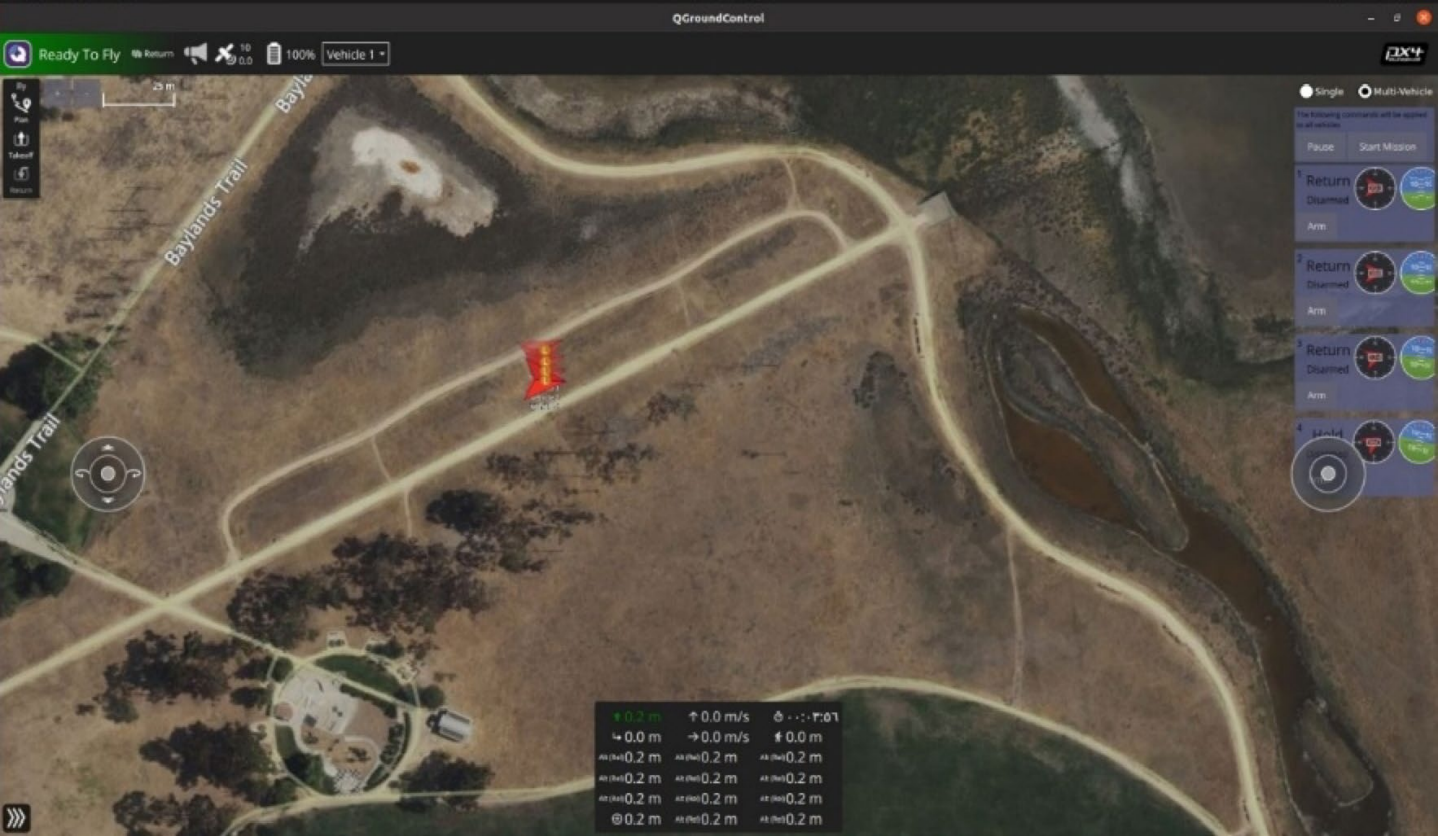
The QGroundControl interface shows real-time telemetry, drone status, and mission progression.

To visualize the mission in real time, the system integrated both the Gazebo simulation and the QGroundControl (QGC) interface. Gazebo provided a 3D view of the drone’s behavior and the search area grid, allowing for the inspection of drone motion, spacing, and environmental interactions. QGroundControl, connected via MAVLink, displayed telemetry for each drone, including GPS position, altitude, speed, and heading. It was instrumental for real-time mission validation, debugging, and verifying the correct execution of waypoint assignments. As illustrated in Fig. 5, the QGroundControl interface provides a comprehensive real-time overview of the swarm’s activity, including UAV locations, mission progress, and telemetry streams. This interface was instrumental in confirming correct path execution, checking drone health, and ensuring synchronized swarm coordination during the high-altitude search mission.

### Evaluation metrics

The evaluation focused on four key performance metrics: Coverage Ratio, Redundancy Ratio, Inter-Drone Distance, and Total Path Length, each assessed over time to offer comprehensive insights into search efficiency and swarm coordination.

The time-based coverage ratio, redundancy ratio, inter-drone distance, and path length metrics (Eqs. 4–7) are adapted and reformulated from prior multi-UAV exploration frameworks^69,70^ Tailored to suit our grid-based, high-altitude thermal search scenario. These formulations provide a structured means to evaluate search efficiency, redundancy avoidance, and swarm coordination. Additionally, a composite Exploration Score was proposed in this work to jointly reflect swarm efficiency, redundancy minimization, and spatial dispersion.

1. **Time-Based Coverage Ratio** $\:\boldsymbol{C}\left(\boldsymbol{t}\right)$: This metric uses (5) to quantify the proportion of the search area that has been uniquely explored by the UAV swarm up to a specific time$\:\:\boldsymbol{t}$. A higher C(t) at earlier time steps shows more efficient exploration behavior.

5 $$\:\boldsymbol{C}\left(\boldsymbol{t}\right)=\:\frac{{\boldsymbol{N}}_{\boldsymbol{u}\boldsymbol{n}\boldsymbol{i}\boldsymbol{q}\boldsymbol{u}\boldsymbol{e}}\left(\boldsymbol{t}\right)}{{\boldsymbol{N}}_{\boldsymbol{t}\boldsymbol{o}\boldsymbol{t}\boldsymbol{a}\boldsymbol{l}}}$$

Where $\:\boldsymbol{C}\left(\boldsymbol{t}\right)$ is the cumulative coverage ratio at time t, $\:{\boldsymbol{N}}_{\boldsymbol{u}\boldsymbol{n}\boldsymbol{i}\boldsymbol{q}\boldsymbol{u}\boldsymbol{e}}\left(\boldsymbol{t}\right)$ Number of uniquely visited grid cells up to time t, $\:{\boldsymbol{N}}_{\boldsymbol{t}\boldsymbol{o}\boldsymbol{t}\boldsymbol{a}\boldsymbol{l}}$ Is the Total number of cells in the grid.

2. **Time-Based Redundancy Ratio**: This metric uses (6) to compare the total number of cell visits (including repeated ones) against the number of unique cells visited by time $\:\boldsymbol{t}$. A low redundancy ratio suggests efficient coverage, while a rising $\:\boldsymbol{R}\left(\boldsymbol{t}\right)$ Shows that drones are increasingly revisiting already explored areas. A value of $\:\boldsymbol{R}=0$ Shows perfect efficiency.

6 $$\:\mathrm{R}\left(\boldsymbol{t}\right)=\:\frac{{\boldsymbol{N}}_{\boldsymbol{t}\boldsymbol{o}\boldsymbol{t}\boldsymbol{a}{\boldsymbol{l}}_{\boldsymbol{v}\boldsymbol{i}\boldsymbol{s}\boldsymbol{i}\boldsymbol{t}\boldsymbol{e}\boldsymbol{d}}}\left(\boldsymbol{t}\right)-{\boldsymbol{N}}_{\boldsymbol{u}\boldsymbol{n}\boldsymbol{i}\boldsymbol{q}\boldsymbol{u}\boldsymbol{e}}\left(\boldsymbol{t}\right)}{{\boldsymbol{N}}_{\boldsymbol{u}\boldsymbol{n}\boldsymbol{i}\boldsymbol{q}\boldsymbol{u}\boldsymbol{e}}}$$

Where R$\:\left(\boldsymbol{t}\right)$ Is the redundancy ratio, $\:{\boldsymbol{N}}_{\boldsymbol{u}\boldsymbol{n}\boldsymbol{i}\boldsymbol{q}\boldsymbol{u}\boldsymbol{e}}\left(\boldsymbol{t}\right)$ is the number of uniquely visited grid cells up to time t and $\:{\boldsymbol{N}}_{\boldsymbol{t}\boldsymbol{o}\boldsymbol{t}\boldsymbol{a}\boldsymbol{l}\_\boldsymbol{v}\boldsymbol{i}\boldsymbol{s}\boldsymbol{i}\boldsymbol{t}\boldsymbol{e}\boldsymbol{d}}$ Is the total number of all visits (including repeats).

3. **Time-Based Average Inter-Drone Distance**: This metric evaluates the average pairwise geodesic distance between drones at each time step $\:\boldsymbol{t}$ Based on (7), it serves as an indicator of the spatial distribution or dispersion of the fleet.

7 $$\:{\boldsymbol{D}}_{\boldsymbol{a}\boldsymbol{v}\boldsymbol{g}}\left(\boldsymbol{t}\right)=\:\frac{1}{\left(\genfrac{}{}{0pt}{}{\boldsymbol{n}}{2}\right)}{\sum\:}_{\boldsymbol{i}=1}^{\boldsymbol{n}}{\sum\:}_{\boldsymbol{j}=1+\boldsymbol{i}}^{\boldsymbol{n}}\boldsymbol{d}({\boldsymbol{p}}_{\boldsymbol{i}}\left(\boldsymbol{t}\right),\:{\boldsymbol{p}}_{\boldsymbol{j}}\left(\boldsymbol{t}\right))$$

Where $\:\boldsymbol{d}({\boldsymbol{p}}_{\boldsymbol{i}}\left(\boldsymbol{t}\right),\:{\boldsymbol{p}}_{\boldsymbol{j}}\left(\boldsymbol{t}\right)\mathrm{):\:is\:the\:distance\:between\:consecutive\:positions}$, $\:{\boldsymbol{D}}_{\boldsymbol{a}\boldsymbol{v}\boldsymbol{g}}\left(\boldsymbol{t}\right):$ It is the average of pairwise distances between all drone positions at a given time.

4. **Total Path Length (L)**: This metric reflects the operational cost of exploration in terms of energy consumption, flight endurance, and mission effort based on (8). While higher path length may imply broader area traversal, it must be interpreted in conjunction with coverage and redundancy to assess overall efficiency.
  8 $$\:\boldsymbol{L}=\:{\sum\:}_{\boldsymbol{i}=1}^{\:\boldsymbol{N}}{\sum\:}_{\boldsymbol{t}=1}^{\boldsymbol{T}-1}\boldsymbol{d}\left({\boldsymbol{p}}_{\boldsymbol{t}}^{\boldsymbol{i}},\:{\boldsymbol{p}}_{\boldsymbol{t}+1}^{\boldsymbol{i}}\right)$$

Where $\:\boldsymbol{d}({\boldsymbol{p}}_{\boldsymbol{t}}^{\boldsymbol{i}},\:{\boldsymbol{p}}_{\boldsymbol{t}+1}^{\boldsymbol{i}}\mathrm{)\:is\:distance\:between\:consecutive\:positions,\:}{\boldsymbol{p}}_{\boldsymbol{t}}^{\boldsymbol{i}}\:\mathbf{i}\mathbf{s}$ Position of the drone $\:\boldsymbol{i}$ at time $\:\boldsymbol{t}$.

5. **Exploration score (E)**: To quantitatively evaluate exploration efficiency in multi-drone systems, we introduce a novel exploration score (9) that integrates coverage ratio, redundancy penalty, and inter-drone spatial dispersion. Unlike prior works that analyze these metrics in isolation, our equation provides a unified, normalized score suitable for algorithm comparison and real-time feedback. The purpose of the Equation is to quantitatively measure how well an optimization algorithm performs exploration — i.e., how effectively it is:

- Covers new areas.
- Avoids redundancy.
- Spreads the drones to search broadly.

9 $$\:\boldsymbol{E}=\:{\boldsymbol{w}}_{1}\boldsymbol{C}+{\boldsymbol{w}}_{2}\left(\frac{1}{\boldsymbol{R}}\right)+{\boldsymbol{w}}_{3}\left(\frac{{\boldsymbol{D}}_{\boldsymbol{a}\boldsymbol{v}\boldsymbol{g}}}{{\boldsymbol{D}}_{\boldsymbol{m}\boldsymbol{a}\boldsymbol{p}}}\right)$$

The coverage ratio (𝐶) quantifies the proportion of previously unexplored cells visited by the swarm, reflecting the efficiency of area exploration. The redundancy ratio (𝑅) denotes the fraction of revisited cells and is inversely weighted within the score to penalize redundant trajectories. The normalized inter-drone distance (𝐷) represents the mean pairwise separation among UAVs, normalized by the maximum theoretical spacing within the search map (𝐷_map_), corresponding to the map diagonal. This term characterizes spatial dispersion, with higher values indicating improved swarm distribution and reduced overlap between sensor footprints.

## Results and discussion

**Fig. 6:**
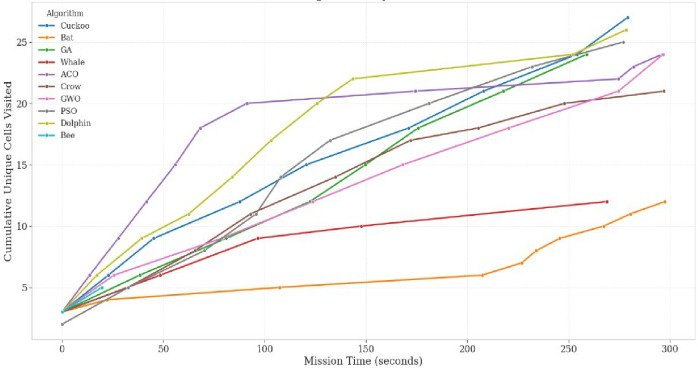
Time-Based Coverage Ratio.

**Fig. 7:**
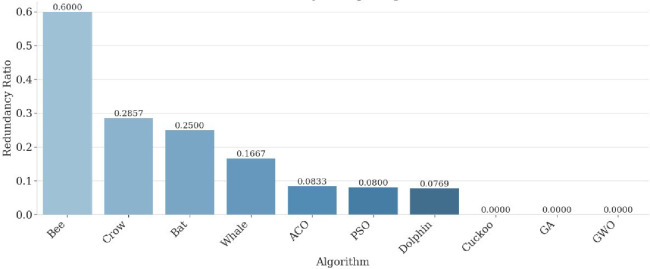
Exploration score per algorithm.

The performance of the proposed multi-drone thermal search system was assessed in a high-altitude PX4 + Gazebo simulation using ten bio-inspired optimization algorithms. A 2D grid-based terrain was constructed, with each cell sized at 30 × 60 m, according to the UAV’s field of view at a 25 m altitude. The evaluation considered four key performance indicators (Coverage Ratio, Redundancy Ratio, Inter-Drone Distance, and Total Path Length) along with the novel Exploration Score (E) proposed in this study. The time-based coverage ratio (Fig. 6) showed that PSO achieved 80% area coverage within 60% of the mission duration, outperforming other algorithms such as GWO and ACO, which needed 75–90% of the mission time for comparable coverage. This efficiency reflects PSO’s velocity-update mechanism, which balances individual and collective intelligence, enabling drones to prioritize unexplored areas. In contrast, GA and CS showed slower convergence, with exploration scores below 0.50, due to revisits during mutation phases (GA) or stochastic Lévy flights (CS). The exploration score results (Fig. 7) confirmed PSO’s superiority, achieving the highest value of 0.67, followed by GWO (0.62) and ACO (0.59). These findings highlight PSO’s effectiveness in balancing rapid area coverage with redundancy avoidance, making it especially suitable for time-sensitive SAR missions.

**Fig. 8:**
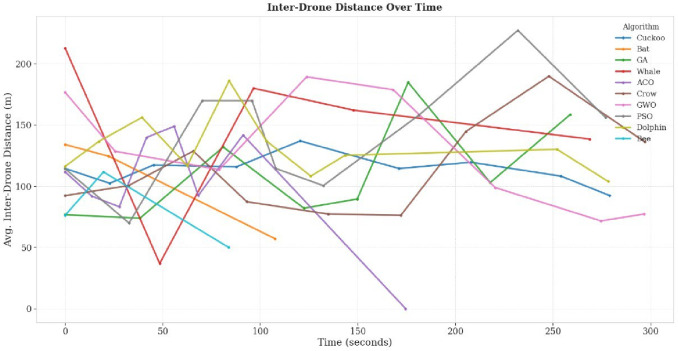
Time-based redundancy ratio.

Figure 8 illustrates the redundancy ratio across algorithms. PSO maintained a low redundancy ratio of approximately 0.25, while ACO and WOA exceeded 0.35, reflecting their tendency to revisit already explored cells. This behavior is consistent with their biological metaphors (pheromone trail reinforcement in ACO and spiral bubble-net movements in WOA), both of which favor local exploitation. The relatively lower exploration performance of ACO can be attributed to the absence of a problem-specific heuristic term. In classical ACO formulations, this heuristic component guides agents toward promising regions and sustains exploration diversity. Its omission in this comparative framework, which prioritized algorithmic consistency over domain-specific tuning, naturally led to stronger exploitation behavior. Conversely, PSO’s distributed update rules ensured a more efficient spread of agents across the grid, reducing overlap and accelerating detection probability.

**Fig. 9:**
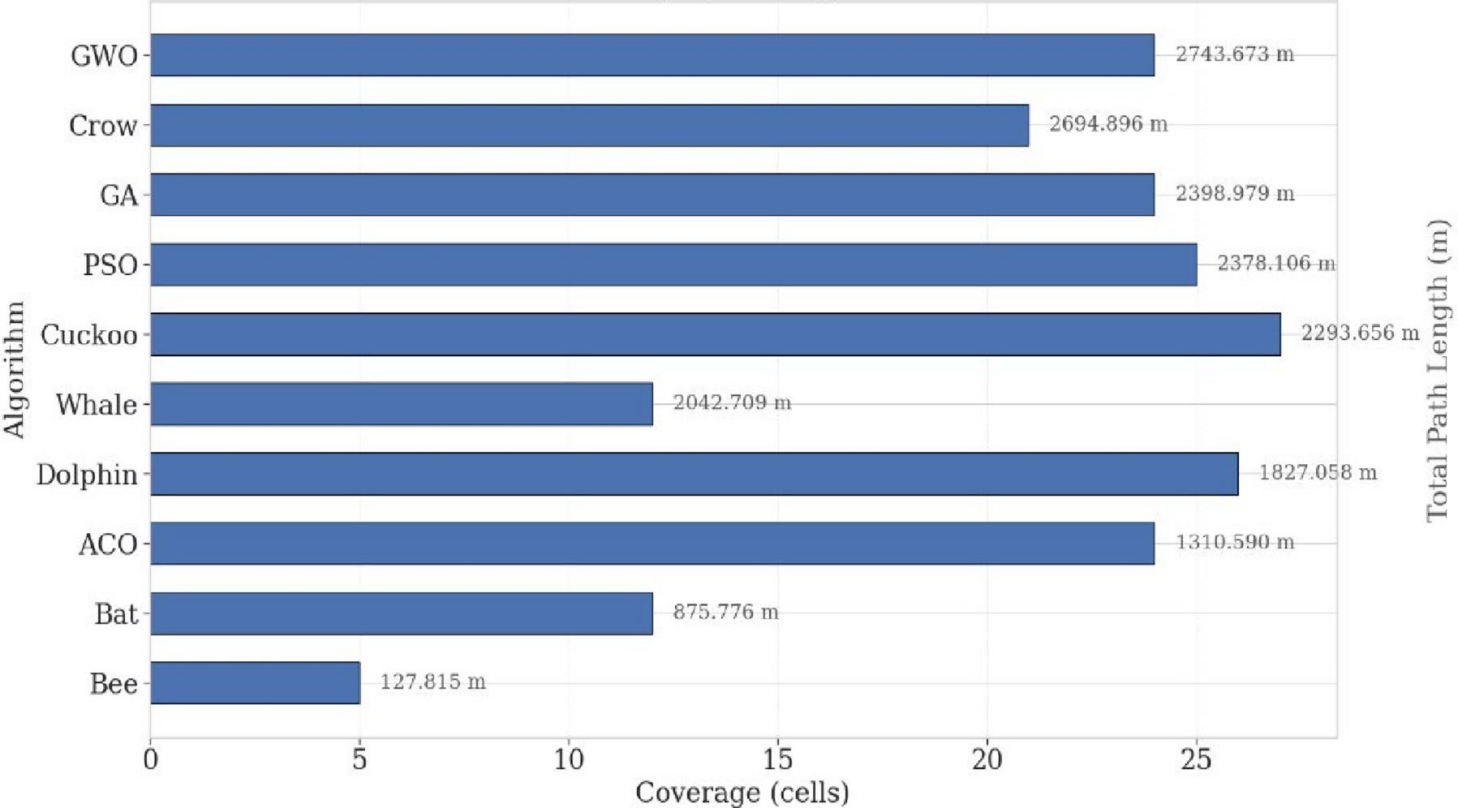
Inter-drone separation.

Maintaining the optimal spatial distribution is crucial for effective collaborative UAV search. Figure 9 shows that PSO sustained an inter-drone separation between 15 and 20 m, preventing both excessive clustering and wide dispersion. This distribution allowed drones to maximize area coverage without leaving gaps. In contrast, WOA occasionally caused UAV clustering during its spiral phase, while GA produced irregular spacing due to stochastic mutations. These behaviors directly affected redundancy and coverage, proving the link between swarm spacing and mission efficiency.

**Fig. 10:**
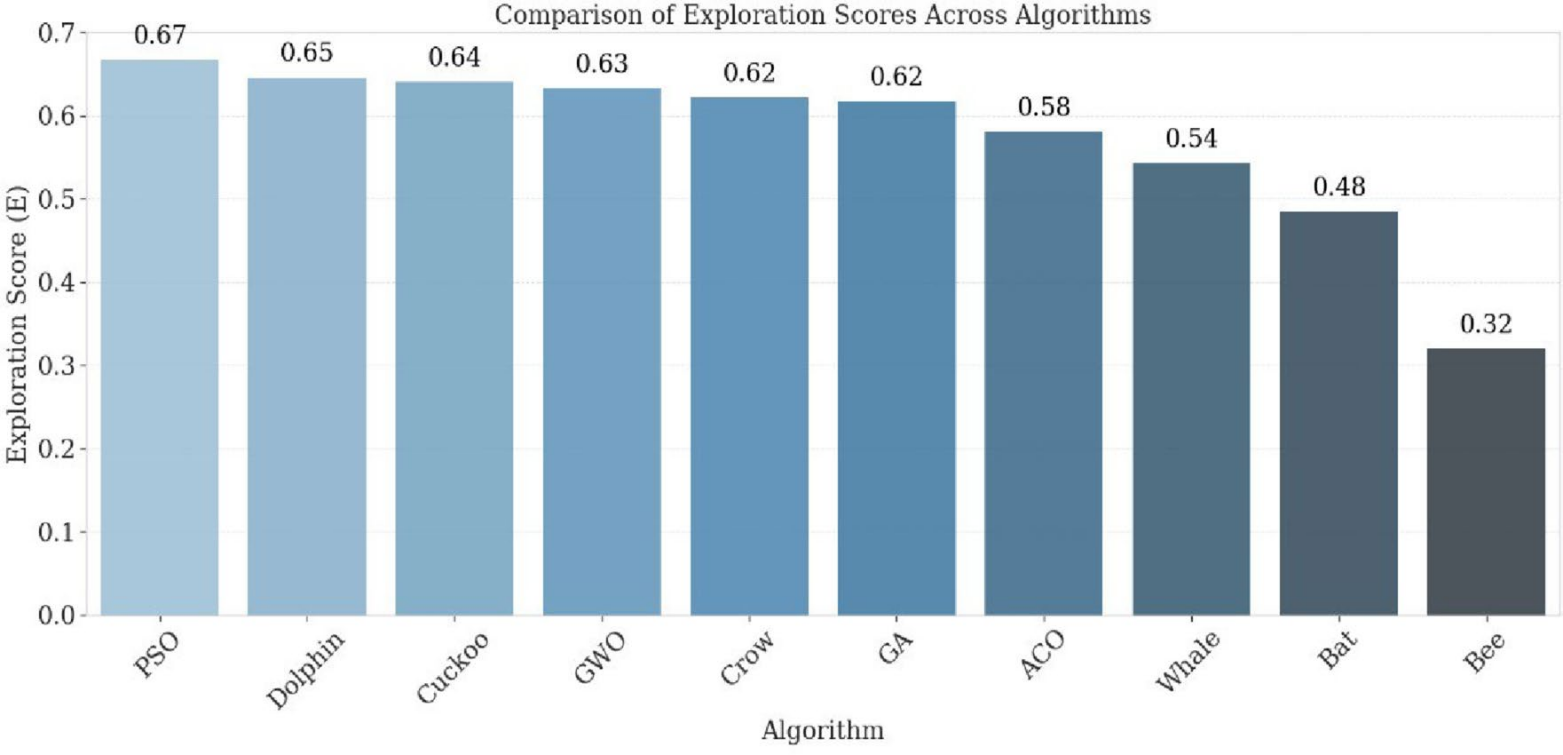
Coverage vs. path length.

Figure 10 compares the total path length to the achieved coverage. Dolphin Optimization Algorithm. Produced the shortest cumulative path length, showing strong energy efficiency; however, its coverage ratio remained below 65%, limiting mission success. PSO generated longer paths but balanced this with broader and faster coverage, achieving a favorable trade-off between energy consumption and mission completeness. This trade-off is particularly relevant for real-world deployments, where UAVs typically have 20–30 min of flight time, needing strategies that prioritize detection probability over minimal travel distance.

A central contribution of this study is the introduction of the Exploration Score (E), a composite performance index that unifies the key aspects of swarm behavior—coverage efficiency, redundancy minimization, and spatial dispersion—into a single normalized measure (8). Unlike conventional analyses that assess these indicators independently, 𝐸 provides a holistic performance criterion that jointly reflects both efficiency and cooperation among UAVs during exploration.

As illustrated in Fig. 7, the comparative results across all algorithms demonstrate that PSO consistently achieved the highest Exploration Score, followed by GWO and ACO, confirming its superior balance between exploration and exploitation. In contrast, stochastic or highly random methods such as Cuckoo Search and the Bee Algorithm exhibited lower 𝐸 values due to redundant cell revisits and uneven coverage.

The use of 𝐸 not only strengthens the fairness of multi-criteria evaluation but also establishes a standardized benchmark for future search-and-rescue research. By condensing mission success into a single interpretable value, the Exploration Score facilitates real-time algorithm assessment, adaptive mission control, and cross-study comparability across diverse UAV coordination frameworks.

## Conclusion

The proposed multi-drone thermal search framework proves significant advancements in enhancing search and rescue operations within hazardous environments. By integrating bio-inspired optimization algorithms with swarm intelligence and real-time thermal detection, the system effectively addresses key challenges, including maximizing area coverage, reducing redundancy in search paths, and enhancing responsiveness to thermal cues. The results reveal that Particle Swarm Optimization (PSO) offers the most effective trade-off, achieving the highest exploration score and highlighting strong coordination among drones during high-altitude search operations. The introduction of a novel exploration score metric further enriches the evaluation of multi-drone search efficiency by jointly considering coverage ratio, redundancy penalties, and spatial distribution of the swarm. The real-time thermal detection pipeline, combined with a grid-based collaborative representation, enables drones to dynamically adapt their paths based on live thermal observations, increasing the chances of prompt survivor detection. The simulation results, confirmed in a Gazebo and PX4 environment, verify the practicality and scalability of the proposed approach. It is essential to note that the proposed framework employs a coverage-based search strategy, concentrating on static, victim-suspected regions within the operational area. The approach is not intended for probabilistic or moving-target scenarios, where temporal dynamics and target mobility must be explicitly modeled. It should be noted that the current framework assumes all UAVs fly at the same altitude without an explicit collision avoidance routine. This simplification enables a clear assessment of algorithmic coordination, but it constrains applicability to open-area, high-altitude searches. Future work will integrate altitude-layer management and reactive avoidance modules to enable fully three-dimensional, collision-aware swarm deployment. Additionally, while the proposed framework demonstrates robust performance in high-altitude thermal-imaging simulations, the current evaluation remains limited to a controlled virtual environment, lacking validation in low-altitude or real-world settings. Future work will extend the system to multi-altitude missions with onboard thermal cameras for detailed inspections, integrate heterogeneous rescue teams that include cooperative ground robots, and perform full-scale field experiments in dynamic disaster scenarios to validate robustness and scalability. In addition, future research may incorporate reinforcement learning for adaptive policy optimization and distributed cooperative control strategies to enhance coordination efficiency and decision-making autonomy in large-scale multi-robot SAR operations. This research contributes a scalable, adaptive, and efficient approach for autonomous SAR operations, highlighting the role of bio-inspired optimization in driving intelligent and collaborative robotic search solutions.

## Data Availability

The datasets used and/or analyzed during the current study are available from the corresponding author on reasonable request.
